# Editorial for the Special Issue on Nanomaterials in Health Care Diagnostics

**DOI:** 10.3390/ma14092214

**Published:** 2021-04-26

**Authors:** Zeynep Altintas

**Affiliations:** Institute of Chemistry, Technical University of Berlin, Straße des 17. Juni 124, 10623 Berlin, Germany; zeynep.altintas@tu-berlin.de

The applications of nanomaterials in health care diagnostics have gained immense interest in recent years owing to their superior properties compared to traditional materials. Many different areas of applications have been identified, spanning from monitoring a disease or treatment to the identification of tissue types in the case of transplant need. Nanomaterials extensively explored for health care diagnostics are predominantly either purely organic or inorganic materials, or a combination of both, i.e., hybrid nanomaterials. Organic nanomaterials, including carbon nanotubes, nanocrystals, liposomes, dendrimers, micelles, hyper-branched organic polymers, molecularly imprinted nanostructures and polymeric hydrogel nanoparticles, have been broadly utilized as imaging and therapeutic agents. Inorganic nanomaterials, such as quantum dots, superparamagnetic iron oxide nanoparticles, metallic nanoparticles, and metal oxides, have also attracted great attention for health care diagnostics, particularly in biosensing and biosensor construction. These materials have critical applications for various molecular imaging techniques including magnetic resonance imaging, positron emission tomography, computed tomography, single-photon emission tomography, optical imaging, and ultrasound imaging techniques.

The unique and admirable characteristics of nanomaterials also allow a wide range of diagnostic approaches, such as surface modification with nanomaterials to obtain more available binding sites for immobilized binding receptor molecules, and signal enhancement using nanoparticle-labelled disease biomarkers to improve sensitivity and specificity of the bio-detection assays. Nanomaterials have been expanding the current situation of molecular diagnostics, point-of-care diagnosis, disease care with therapeutics, and also personalized medicine. The integration of biomarker discovery into the nanodevices, with the combination and modifications of nanomaterials, has improved the clinical and research-based applications for cancer, cardiovascular, infectious and neurological diseases in recent years. The further engineered nanomaterials, in combination with the current developments, will spectacularly impact health care diagnostics and result in synergetic medical solutions.

There are nine papers published in this Special Issue, covering applications of various nanomaterials in health care [1,2,3,4,5,6,7,8,9]. In particular, Savas et al. reported the development of antibody and DNA sensors for Salmonella detection using a custom-made microfluidic-based electrochemical system [1]. *Salmonella typhimurium* from human stool samples as well as Salmonella DNA could be detected with the limit of detection (LOD) of 1 cfu mL^−1^ and 0.94 nM, respectively. Both sensors offer rapid, highly sensitive, and specific diagnostic assay approaches for pathogen detection [1]. Bakhori et al. proposed a surface-enhanced CdSe/ZnS QD/SiNP electrochemical immunosensor for the diagnosis of *Mycobacterium tuberculosis* by the combination of CFP10–ESAT6 for enhanced specificity [2]. The fabricated electrode was linked to the biocatalytic action of the enzyme catalase through antigen–antibody binding for the detection of the antigen (CFP10–ESAT6) by means of producing a differential pulse voltammetry (DPV) current. The modified electrode enhanced electron transfer between the electrode and the analyte. The active surface area was found to be 4.14-fold higher than the bare SPCE. The developed method showed high selectivity towards CFP10–ESAT6 compared with the other tuberculosis proteins. The LOD was determined as 1.5 × 10^−10^ g mL^−1^ for a linear range of 40 to 100 ng mL^−1^ of CFP10–ESAT6 concentration, and the proposed method showed good reproducibility of the target analyte with a relative standard deviation of 1.45% [2].

Mansuriya and Altintas reviewed the graphene quantum dot (GQD)-based electrochemical immunosensors for biomedical applications for the first time in this Special Issue [3]. GQDs have garnered remarkable attention in immunosensor development, owing to their special attributes such as large surface area, excellent biocompatibility, quantum confinement, edge effects, and abundant sites for chemical modification. In addition to these distinct features, GQDs acquire peroxidase (POD)-mimicking electro-catalytic activity, and hence, they can replace horseradish peroxidase (HRP)-based systems to perform facile, quick, and inexpensive label-free immunoassays. In this review, the authors’ main motivation was to summarize and focus on the recent advances in GQD-based electrochemical immunosensors for the early and rapid detection of cancer, cardiovascular disorders, and pathogenic diseases. They also highlighted the underlying principles of electrochemical immunosensing techniques [3]. Savas and Altintas have presented an excellent example for GQD-based electrochemical immunosensors with their work on the electrochemical sensing of *Yersinia enterocolitica* in milk and human serum, where GODs exhibited POD-mimicking behavior and allowed the amperometric detection of the pathogenic bacterium without a need for any enzyme label. The fabricated sensor could detect the analyte in milk and human serum with LODs of 5 cfu mL^−1^ and 30 cfu mL^−1^, respectively [4].

Cao et al. purposed a copper oxide/zinc oxide composite nano-surface for use in biosensors, which were prepared using a simple and inexpensive distributed colloidal technique [5]. They compared the combinations of mixed dispersions with volume ratios of 1:1, 1:2 and 2:1 ZnO:CuO. They analyzed the uniform nano-crystalline sensor surfaces on polyethylene terephthalate using scanning electron microscopy, atomic force microscopy and Raman spectroscopy. The ZnO–CuO composite biosensor nano-surfaces showed a significantly increased impedimetric signal in comparison to pure ZnO nanocrystals, and the maximum output was achieved with a volume ratio of 1:2 ZnO/CuO. Moreover, the antibody capture of C-reactive protein on the nano-surfaces was examined to demonstrate the enhanced signal generated with increasing amounts of CuO on the nano-surface [5].

The quantitative determination of immunoglobulin free light chains (FLCs) is considered to be the gold standard in the diagnosis and treatment of amyloid light-chain (AL) amyloidosis and multiple myeloma (MM). Lizoń et al. studied a silver nanoparticle-based assay for the detection of FLCs [6]. The authors achieved the optimal test conditions when a metal nanoparticle (MNP) was covered with 10 particles of an antibody and conjugated by 5–50 protein antigen particles (FLCs). The parameters of the specific immunochemical aggregation process were found to be consistent with the sizes of AgNPs and the protein particles, which were confirmed by four physical methods, yielding complementary data concerning a clinically useful AgNPs aggregation test [6].

Factor V Leiden (FV Leiden) mutation is a common inherited risk factor predisposing to venous thromboembolism. In their study, Erdem and Eksin aimed to perform the impedimetric detection of the FV Leiden mutation by a zip nucleic acid (ZNA) probe-based assay in combination with carbon nanofibers modified screen-printed electrodes. They also examined the selectivity of the assay against the mutation-free DNA sequences as well as the synthetic PCR samples [7].

Human saliva provides a great opportunity for developing non-invasive diagnostics. It contains naturally occurring nanoparticles with unique biochemical and structural features. The salivary exosome, a nanoscale extracellular vesicle, has been well known as a vastly enlightening nanovesicle with clinically relevant information. Salivary exosomes have brought forth a pathway and mechanism by which cancer-derivative biomarkers can be transported through the systemic circulation into the oral cavity. In their review, Chen et al. provided a current array of discovered salivary biomarkers and nanostructural properties of salivary exosomes associated with specific cancers [8]. In addition, they described a novel electrochemical sensing technology (i.e., electric field-induced release and measurement) that enhances saliva liquid biopsy, covering the current landscape of point-of-care saliva testing [8].

Vancomycin is considered as the treatment of choice for the infections of methicillin-resistant *Staphylococcus aureus* (MRSA). Clinical studies have confirmed that combinations of vancomycin and beta-lactams have improved patient outcomes compared to vancomycin alone for the treatment of MRSA bloodstream infections. Bhise et al. reported a robust method to accomplish high loading of vancomycin and cefazolin (CFZ) in unilamellar liposomes. Liposomal vancomycin (LVAN) reduced minimum inhibitory concentration (MIC) values 2-fold compared to commercial vancomycin. The combination of liposomal vancomycin (LVAN) and liposomal CFZ (LCFZ) verified a 7.9-fold reduction in comparison to LVAN alone. Rhodamine dye-loaded liposomes demonstrated superior cellular uptake in macrophage-like RAW 264.7 cells. The authors concluded that the developed formulations of vancomycin, when administered alone or in combination with CFZ, provide a rational approach for fighting MRSA infections [9].

I would like to take this opportunity to thank all the authors for submitting their papers to this Special Issue. I also want to thank all the reviewers for dedicating their time and helping to improve the quality of the submitted papers.

## Data Availability

Not applicable.
